# 2,4-Dichloro­benzaldehyde

**DOI:** 10.1107/S160053680905435X

**Published:** 2009-12-24

**Authors:** Ricardo Cabello, Maksymilian Chruszcz, Wladek Minor

**Affiliations:** a University of Virginia, Department of Molecular Physiology & Biological Physics, 1340 Jefferson Park Avenue, Charlottesville, VA 22908, USA

## Abstract

In the crystal structure of the title compound, C_7_H_4_Cl_2_O, the mol­ecules form a network of weak C—H⋯O inter­actions involving the aldehyde O atom and the *ortho*-H atom on the benzene ring together with C—H⋯O inter­actions between the formyl groups.  Together, these connect the mol­ecules into (10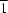) layers, which are stabilized additionally by π–π stacking inter­actions of the benzene rings [centroid–centroid distance = 3.772 (1) Å]. The aldehyde group is twisted relative to the benzene ring by 7.94 (13)°.

## Related literature

For applications of the title compound, see: Katagi (1988 ▶); Wang *et al.* (2004 ▶). For a related structure, see: Gawlicka-Chruszcz *et al.* (2006 ▶).
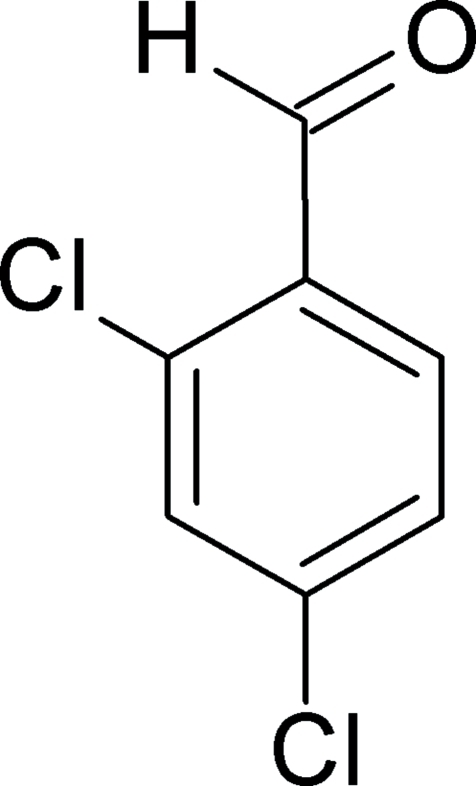


## Experimental

### 

#### Crystal data

C_7_H_4_Cl_2_O
*M* *_r_* = 175.01
Monoclinic, 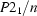

*a* = 13.100 (1) Å
*b* = 3.772 (1) Å
*c* = 15.332 (1) Å
β = 113.797 (2)°
*V* = 693.2 (3) Å^3^
*Z* = 4
Mo *K*α radiation
μ = 0.85 mm^−1^
*T* = 100 K
0.40 × 0.10 × 0.10 mm

#### Data collection

Rigaku R-AXIS RAPID diffractometer
Absorption correction: multi-scan (Otwinowski *et al.*, 2003 ▶) *T*_min_ = 0.90, *T*_max_ = 0.92
6924 measured reflections
3737 independent reflections
3221 reflections with *I* > 2σ(*I*)
*R*_int_ = 0.063

#### Refinement

*R*[*F* ^2^ > 2σ(*F* ^2^)] = 0.036
*wR*(*F* ^2^) = 0.114
*S* = 1.10
3737 reflections
107 parameters
All H-atom parameters refined
Δρ_max_ = 0.67 e Å^−3^
Δρ_min_ = −0.41 e Å^−3^

### 

Data collection: *HKL-2000* (Otwinowski & Minor, 1997 ▶); cell refinement: *HKL-2000*; data reduction: *HKL-2000*; program(s) used to solve structure: *SHELXS97* (Sheldrick, 2008 ▶) and *HKL-3000SM* (Minor *et al.*, 2006 ▶); program(s) used to refine structure: *SHELXL97* (Sheldrick, 2008 ▶) and *HKL-3000SM*; molecular graphics: *HKL-3000SM*, *ORTEPIII* (Burnett & Johnson, 1996 ▶), *ORTEP-3* (Farrugia, 1997 ▶), *Mercury* (Macrae *et al.*, 2006 ▶) and *POV-RAY* (The *POV-RAY* Team, 2004 ▶); software used to prepare material for publication: *HKL-3000SM*.

## Supplementary Material

Crystal structure: contains datablocks I, global. DOI: 10.1107/S160053680905435X/gk2246sup1.cif

Structure factors: contains datablocks I. DOI: 10.1107/S160053680905435X/gk2246Isup2.hkl

Additional supplementary materials:  crystallographic information; 3D view; checkCIF report

## Figures and Tables

**Table 1:** Hydrogen-bond geometry (Å, °)

*D*—H⋯*A*	*D*—H	H⋯*A*	*D*⋯*A*	*D*—H⋯*A*
C7—H7⋯O1^i^	0.946 (17)	2.533 (17)	3.4289 (11)	158.0 (14)
C6—H6⋯O1^ii^	0.950 (19)	2.512 (17)	3.2774 (11)	137.8 (12)

Symmetry codes: (i) 
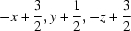
; (ii) 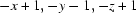.

**Fig. 1.**
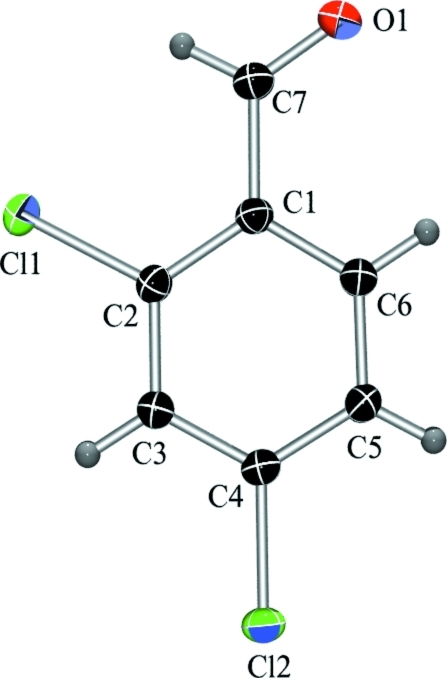
The asymmetric unit of the reported structure. Displacement ellipsoids are drawn at the 50% probability level and hydrogen atoms are drawn as grey spheres of an arbitrary radius.

**Fig. 2.**
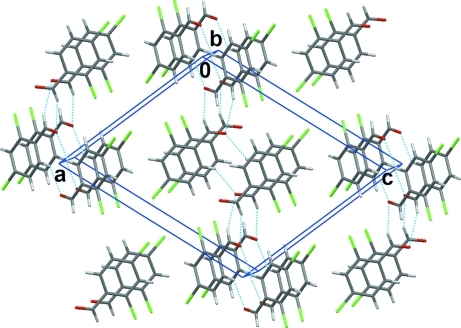
The molecular packing of 2,4-dichlorobenzaldehyde. Weak interactions, in which the oxygen atom participates, are shown as blue, dashed lines.

## supplementary crystallographic information

### Comment

2,4-Dichlorobenzaldehyde is primarily used in the preparation of dyes, insecticides, herbicides, antiseptics and disinfectants (Wang *et al.*, 2004). It is also used as an intermediate of organic synthesis of fungicide diniconazole (Katagi, 1988).

In the crystal structure of 2,4-dichlorobenzaldehyde (Fig. 1), the aldehyde group is twisted relative to the benzene ring with torsion angles C6—C1—C7—O1 and C2—C1—C7—O1 being -7.94 (13)° and 170.86 (9)°. These torsion angles are significantly smaller in comparison to the corresponding angles in 2,6-dichlorobenzaldehyde (Gawlicka-Chruszcz *et al.*, 2006) which are -27.3° and 152.6° respectively. Significantly bigger twist of the aldehyde group in the case of 2,6-dichlorobenzaldehyde is caused by presence of the chlorine atoms in ortho positions.

The change of the position of chlorine atom causes that interactions in which chlorine atoms are involved in 2,4-dichlorobenzaldehyde and 2,6-dichlorobenzaldehyde differ significantly. In the case of 2,6-dichlorobenzaldehyde Cl2 was involved in weak interaction with hydrogen atom from neighboring benzene ring, while in 2,4-dichlorobenzaldehyde structure such interactions are not observed for any of the chlorine atoms. However, in the case of 2,4-dichlorobenzaldehyde, the chlorine atoms from neighboring molecules form short contacts with Cl1···Cl2 (1/2 + *x*,1/2 - *y*,1/2 + *z*) distance being 3.442Å (Fig. 2).

The weak O···H—C interactions (Table 1) between the aldehyde oxygen and the benzene hydrogen atoms connect molecules to form layers, which are additionally stabilized by stacking of benzene rings (Fig. 2). The oxygen atom from the aldehyde group plays a central role in the formation of weak interactions, and O1···H6—C6 (1 - *x*,-1 - *y*,1 - *z*) and O1···H7—C7 (1,5 - *x*,-1/2 + *y*,1.5 - *z*) distances are 2.51Å and 2.53Å respectively.

### Experimental

2,4-dichlorobenzaldehyde was purchased from ALDRICH (99% purity, lot 08722CD). The compound was provided in crystalline form.

### Refinement

All hydrogen atoms were localized using the difference density Fourier map. Their positions and isotropic displacement parameters were refined.

### Crystal data

**Table:**

C_7_H_4_Cl_2_O	*F*(000) = 352
*M**_r_* = 175.01	*D*_x_ = 1.677 Mg m^−^^3^
Monoclinic, *P*2_1_/*n*	Mo *K*α radiation, λ = 0.71074 Å
Hall symbol: -P 2yn	Cell parameters from 31891 reflections
*a* = 13.100 (1) Å	θ = 1.0–37.8°
*b* = 3.772 (1) Å	µ = 0.85 mm^−^^1^
*c* = 15.332 (1) Å	*T* = 100 K
β = 113.797 (2)°	Block, colorless
*V* = 693.2 (3) Å^3^	0.40 × 0.10 × 0.10 mm
*Z* = 4	

### Data collection

**Table:**

Rigaku R-AXIS RAPID diffractometer	3737 independent reflections
Radiation source: fine-focus sealed tube	3221 reflections with *I* > 2σ(*I*)
graphite	*R*_int_ = 0.063
Detector resolution: 10 pixels mm^-1^	θ_max_ = 37.8°, θ_min_ = 1.0°
ω scans	*h* = −22→22
Absorption correction: multi-scan (Otwinowski *et al.*, 2003)	*k* = −6→6
*T*_min_ = 0.90, *T*_max_ = 0.92	*l* = −26→24
6924 measured reflections	

### Refinement

**Table:**

Refinement on *F*^2^	Primary atom site location: structure-invariant direct methods
Least-squares matrix: full	Secondary atom site location: difference Fourier map
*R*[*F*^2^ > 2σ(*F*^2^)] = 0.036	Hydrogen site location: difference Fourier map
*wR*(*F*^2^) = 0.114	All H-atom parameters refined
*S* = 1.10	*w* = 1/[σ^2^(*F*_o_^2^) + (0.0708*P*)^2^ + 0.0197*P*] where *P* = (*F*_o_^2^ + 2*F*_c_^2^)/3
3737 reflections	(Δ/σ)_max_ = 0.001
107 parameters	Δρ_max_ = 0.67 e Å^−^^3^
0 restraints	Δρ_min_ = −0.41 e Å^−^^3^

### Special details

**Table:**

Geometry. All e.s.d.'s (except the e.s.d. in the dihedral angle between two l.s. planes) are estimated using the full covariance matrix. The cell e.s.d.'s are taken into account individually in the estimation of e.s.d.'s in distances, angles and torsion angles; correlations between e.s.d.'s in cell parameters are only used when they are defined by crystal symmetry. An approximate (isotropic) treatment of cell e.s.d.'s is used for estimating e.s.d.'s involving l.s. planes.
Refinement. Refinement of *F*^2^ against ALL reflections. The weighted *R*-factor *wR* and goodness of fit *S* are based on *F*^2^, conventional *R*-factors *R* are based on *F*, with *F* set to zero for negative *F*^2^. The threshold expression of *F*^2^ > σ(*F*^2^) is used only for calculating *R*-factors(gt) *etc*. and is not relevant to the choice of reflections for refinement. *R*-factors based on *F*^2^ are statistically about twice as large as those based on *F*, and *R*- factors based on ALL data will be even larger.

### Fractional atomic coordinates and isotropic or equivalent isotropic displacement parameters (Å^2^)

**Table:**

	*x*	*y*	*z*	*U*_iso_*/*U*_eq_	
Cl2	0.138241 (15)	0.17792 (6)	0.579292 (15)	0.02422 (7)	
Cl1	0.558151 (16)	0.15135 (6)	0.840216 (14)	0.02526 (7)	
C1	0.49878 (6)	−0.1166 (2)	0.66074 (6)	0.01968 (13)	
C3	0.35318 (6)	0.1385 (2)	0.70157 (6)	0.02006 (14)	
C2	0.46410 (6)	0.0484 (2)	0.72565 (5)	0.01980 (13)	
C6	0.41880 (6)	−0.1866 (2)	0.56890 (6)	0.02060 (14)	
C5	0.30765 (6)	−0.0964 (2)	0.54240 (6)	0.02080 (13)	
C4	0.27652 (6)	0.0634 (2)	0.60987 (5)	0.01992 (13)	
O1	0.64561 (5)	−0.4059 (2)	0.63526 (5)	0.02975 (14)	
C7	0.61622 (6)	−0.2245 (2)	0.68671 (6)	0.02340 (14)	
H3	0.3319 (13)	0.260 (4)	0.7450 (12)	0.038 (3)*	
H5	0.2576 (13)	−0.150 (4)	0.4813 (13)	0.042 (4)*	
H6	0.4423 (13)	−0.293 (5)	0.5239 (12)	0.046 (4)*	
H7	0.6686 (14)	−0.131 (4)	0.7449 (13)	0.041 (4)*	

### Atomic displacement parameters (Å^2^)

**Table:**

	*U*^11^	*U*^22^	*U*^33^	*U*^12^	*U*^13^	*U*^23^
Cl2	0.01697 (10)	0.02944 (12)	0.02558 (11)	0.00285 (6)	0.00789 (8)	0.00236 (6)
Cl1	0.02130 (11)	0.02926 (12)	0.02104 (11)	−0.00059 (6)	0.00422 (8)	−0.00518 (6)
C1	0.0165 (3)	0.0207 (3)	0.0213 (3)	−0.0005 (2)	0.0071 (2)	−0.0007 (2)
C3	0.0190 (3)	0.0209 (3)	0.0206 (3)	0.0003 (2)	0.0083 (3)	−0.0003 (2)
C2	0.0182 (3)	0.0203 (3)	0.0195 (3)	−0.0014 (2)	0.0062 (2)	−0.0017 (2)
C6	0.0189 (3)	0.0226 (3)	0.0206 (3)	−0.0002 (2)	0.0082 (2)	−0.0008 (2)
C5	0.0185 (3)	0.0229 (3)	0.0195 (3)	−0.0007 (2)	0.0061 (2)	−0.0008 (2)
C4	0.0168 (3)	0.0209 (3)	0.0215 (3)	0.0001 (2)	0.0071 (2)	0.0016 (2)
O1	0.0214 (3)	0.0378 (3)	0.0304 (3)	0.0045 (2)	0.0108 (2)	−0.0046 (3)
C7	0.0178 (3)	0.0266 (3)	0.0251 (3)	−0.0001 (3)	0.0080 (3)	−0.0013 (3)

### Geometric parameters (Å, °)

**Table:**

Cl2—C4	1.7327 (7)	C3—H3	0.939 (17)
Cl1—C2	1.7343 (8)	C6—C5	1.3869 (11)
C1—C2	1.3961 (11)	C6—H6	0.950 (19)
C1—C6	1.3999 (11)	C5—C4	1.3930 (11)
C1—C7	1.4820 (11)	C5—H5	0.923 (18)
C3—C4	1.3877 (11)	O1—C7	1.2180 (11)
C3—C2	1.3893 (11)	C7—H7	0.946 (17)
			
C2—C1—C6	118.32 (7)	C1—C6—H6	118.6 (9)
C2—C1—C7	122.14 (7)	C6—C5—C4	118.43 (7)
C6—C1—C7	119.53 (7)	C6—C5—H5	118.4 (10)
C4—C3—C2	118.11 (7)	C4—C5—H5	123.2 (10)
C4—C3—H3	121.2 (10)	C3—C4—C5	122.11 (7)
C2—C3—H3	120.6 (10)	C3—C4—Cl2	118.26 (6)
C3—C2—C1	121.73 (7)	C5—C4—Cl2	119.62 (6)
C3—C2—Cl1	116.99 (6)	O1—C7—C1	123.05 (8)
C1—C2—Cl1	121.28 (6)	O1—C7—H7	121.4 (10)
C5—C6—C1	121.30 (7)	C1—C7—H7	115.5 (10)
C5—C6—H6	120.0 (9)		
			
C4—C3—C2—C1	−0.72 (12)	C1—C6—C5—C4	−0.68 (12)
C4—C3—C2—Cl1	179.11 (6)	C2—C3—C4—C5	−0.19 (12)
C6—C1—C2—C3	0.90 (12)	C2—C3—C4—Cl2	−179.59 (6)
C7—C1—C2—C3	−177.92 (8)	C6—C5—C4—C3	0.88 (12)
C6—C1—C2—Cl1	−178.92 (6)	C6—C5—C4—Cl2	−179.73 (6)
C7—C1—C2—Cl1	2.26 (11)	C2—C1—C7—O1	170.86 (9)
C2—C1—C6—C5	−0.18 (12)	C6—C1—C7—O1	−7.94 (13)
C7—C1—C6—C5	178.67 (7)		

### Hydrogen-bond geometry (Å, °)

**Table:**

*D*—H···*A*	*D*—H	H···*A*	*D*···*A*	*D*—H···*A*
C7—H7···O1^i^	0.946 (17)	2.533 (17)	3.4289 (11)	158.0 (14)
C6—H6···O1^ii^	0.950 (19)	2.512 (17)	3.2774 (11)	137.8 (12)

Symmetry codes: (i) −*x*+3/2, *y*+1/2, −*z*+3/2; (ii) −*x*+1, −*y*−1, −*z*+1.
